# Supplementary material for: Super-additive cooperation
Source: Nature. 2024 Feb 21;626(8001):1034–41. doi: 10.1038/s41586-024-07077-w (PMC10901731; doi:10.1038/s41586-024-07077-w)
Supplement: Supplementary file 1 — Reporting Summary [file 41586_2024_7077_MOESM1_ESM.pdf]

nature portfolio

Corresponding author(s): Fehr, Ernst

Last updated by author(s): 25 September 2023

Reporting Summary

Nature Portfolio wishes to improve the reproducibility of the work that we publish. This form provides structure for consistency and transparency in reporting. For further information on Nature Portfolio policies, see our Editorial Policies and the Editorial Policy Checklist.

Statistics

For all statistical analyses, confirm that the following items are present in the figure legend, table legend, main text, or Methods section.

n/a	Confirmed	
☐	☒	The exact sample size (*n*) for each experimental group/condition, given as a discrete number and unit of measurement
☐	☒	A statement on whether measurements were taken from distinct samples or whether the same sample was measured repeatedly
☐	☒	The statistical test(s) used AND whether they are one- or two-sided<br>*Only common tests should be described solely by name; describe more complex techniques in the Methods section.*
☐	☒	A description of all covariates tested
☐	☒	A description of any assumptions or corrections, such as tests of normality and adjustment for multiple comparisons
☐	☒	A full description of the statistical parameters including central tendency (e.g. means) or other basic estimates (e.g. regression coefficient) AND variation (e.g. standard deviation) or associated estimates of uncertainty (e.g. confidence intervals)
☐	☒	For null hypothesis testing, the test statistic (e.g. *F*, *t*, *r*) with confidence intervals, effect sizes, degrees of freedom and *P* value noted<br>*Give P values as exact values whenever suitable.*
☒	☐	For Bayesian analysis, information on the choice of priors and Markov chain Monte Carlo settings
☒	☐	For hierarchical and complex designs, identification of the appropriate level for tests and full reporting of outcomes
☐	☒	Estimates of effect sizes (e.g. Cohen's *d*, Pearson's *r*), indicating how they were calculated

Our web collection on statistics for biologists contains articles on many of the points above.

Software and code

Policy information about availability of computer code

Data collection	No software was used for collection of experimental data.
Data analysis	Data were analyzed with R (4.1.3).

For manuscripts utilizing custom algorithms or software that are central to the research but not yet described in published literature, software must be made available to editors and reviewers. We strongly encourage code deposition in a community repository (e.g. GitHub). See the Nature Portfolio guidelines for submitting code & software for further information.

Data

Policy information about availability of data

All manuscripts must include a data availability statement. This statement should provide the following information, where applicable:

- Accession codes, unique identifiers, or web links for publicly available datasets
- A description of any restrictions on data availability
- For clinical datasets or third party data, please ensure that the statement adheres to our policy

The experimental data and code for analyses are available at www.github.com/cmefferson/superAdditiveCooperation in the directory "data".

Human research participants

Policy information about studies involving human research participants and Sex and Gender in Research.

Reporting on sex and gender	At the time of data collection, the difference between sex and gender was not a salient distinction among Perepkas and Ngenikas. Before participating in the experiment proper, each participant responded to a short questionnaire. We collected data on the gender/sex of participants at this time. The data are used as controls in statistical analyses and are available with the publicly posted raw data. However, the data are fully anonymized, and individuals are not identifiable. 37% of participants were female.
Population characteristics	See above.
Recruitment	See below under "Sampling strategy" for a complete description of recruitment and sampling. As is typically true for behavioral experiments, recruitment into the study was not representative. Conditional on participating, however, assignment to treatment was random, and assignment to role as Player 1 or Player 2 was random.
Ethics oversight	IRB of the Faculty of Business, Economics and Informatics at the University of Zurich.

Note that full information on the approval of the study protocol must also be provided in the manuscript.

Field-specific reporting

Please select the one below that is the best fit for your research. If you are not sure, read the appropriate sections before making your selection.

☐ Life sciences ☒ Behavioural & social sciences ☐ Ecological, evolutionary & environmental sciences

For a reference copy of the document with all sections, see nature.com/documents/nr-reporting-summary-flat.pdf

Behavioural & social sciences study design

All studies must disclose on these points even when the disclosure is negative.

Study description	The empirical study was a standard behavioral experiment in the tradition of experimental economics (e.g. no deception of participants, choices were incentivized). Specifically, the experiment was a one-shot sequential social dilemma with a continuous action space that is essentially a one-shot symmetric trust game.
Research sample	Experimental subjects were adult Perepkas and adult Ngenikas from the western Highlands of Papua New Guinea. The main paper discusses at length the rationale for using this sample when examining the evolution of cooperation.
Sampling strategy	The sample was a convenience sample. Specifically, Helen Bernhard visited each of the two groups on two separate days to recruit participants, which meant a total of four days recruiting. On a given day, she walked through the settlements and talked to adults present and invited them to the study in a few days time. No statistical methods were used to pre-determine sample size, and practical concerns (e.g., time in the field) were critical. However, with two treatments, our sample size would provide adequate power (alpha = 0.05, beta = 0.8), given an OLS model fully saturated with respect to experimental design, for an approximate effect size associated with R^2 = 0.9, i.e. Cohen's f^2 = 0.111. In practice, this is a lower bound for Players 2 because we have multiple observations per player.
Data collection	Data were recorded with pen and paper. Helen Bernhard conducted the experiment in-person. Her spouse, who does not speak Tok Pisin, was nearby to pay participants their show-up fees and provide participants with refreshments.
Timing	Data were collected in July 2004.
Data exclusions	No data were excluded.
Non-participation	No participants dropped out or declined participation. Some participants could not participate because they did not correctly answer a series of questions that tested comprehension of the game. To proceed to the experiment proper, a person had to answer all of these questions correctly. This was a pre-determined criterion in the following. People who did not meet the criterion received a show-up fee but did not participate in the experiment itself.
Randomization	Treatments and role (Player 1 vs Player 2) were first assigned to specific numbers. Participants were randomly assigned to treatment and role by blindly drawing numbers written on small pieces of paper from a bowl.

Reporting for specific materials, systems and methods

We require information from authors about some types of materials, experimental systems and methods used in many studies. Here, indicate whether each material, system or method listed is relevant to your study. If you are not sure if a list item applies to your research, read the appropriate section before selecting a response.

Materials & experimental systems

n/a	Involved in the study
☒	☐ Antibodies
☒	☐ Eukaryotic cell lines
☒	☐ Palaeontology and archaeology
☒	☐ Animals and other organisms
☒	☐ Clinical data
☒	☐ Dual use research of concern

Methods

n/a	Involved in the study
☒	☐ ChIP-seq
☒	☐ Flow cytometry
☒	☐ MRI-based neuroimaging
